# Mallory-Weiss Syndrome Without Vomiting Following Cardiopulmonary Resuscitation With a Laryngeal Tube

**DOI:** 10.7759/cureus.110809

**Published:** 2026-06-14

**Authors:** Satoshi Homma

**Affiliations:** 1 Department of Emergency and Disaster Medicine, The Jikei University Hospital, The Jikei University School of Medicine, Kashiwa, JPN

**Keywords:** airway management complications, cardiopulmonary resuscitation(cpr), gastric mucosal tear, laryngeal tube, mallory-weiss syndrome, out-of-hospital cardiac arrest, supraglottic airway device, upper gastrointestinal bleeding

## Abstract

Mallory-Weiss syndrome (MWS) is characterized by longitudinal mucosal lacerations at the esophagogastric junction and is an established cause of nonvariceal upper gastrointestinal bleeding (UGIB), classically triggered by forceful vomiting, retching, or coughing. We report a case of MWS occurring after cardiopulmonary resuscitation (CPR) with a laryngeal tube (LT) in the complete absence of antecedent vomiting.

A 77-year-old woman with no history of vomiting, alcohol use, or gastrointestinal disease experienced out-of-hospital cardiac arrest (OHCA). Emergency medical services initiated resuscitation and, after approximately 15 minutes of assisted bag-valve-mask ventilation followed by cardiopulmonary resuscitation, secured the airway with a size-3 LT (Smiths Medical Japan, Tokyo, Japan), a standard LT that lacks a gastric drainage channel. Defibrillation shocks were delivered for ventricular tachycardia, and return of spontaneous circulation (ROSC) was achieved after approximately 29 minutes of CPR. On arrival, the patient was hemodynamically unstable with a blood pressure of 89/56 mmHg and a Glasgow Coma Scale (GCS) score of 3. Contrast-enhanced CT, performed because of unexplained hemodynamic instability, revealed active contrast extravasation into the gastric lumen. A nasogastric (NG) tube subsequently yielded bloody aspirate, and emergent upper gastrointestinal endoscopy identified a longitudinal mucosal laceration at the gastric cardia, confirming MWS. Because CT preceded NG tube insertion, iatrogenic injury from NG tube placement was excluded as the cause of the laceration. Endoscopic hemostasis was achieved, and the patient received four units of packed red blood cells and four units of fresh frozen plasma. Despite successful hemostasis, she developed severe hypoxic-ischemic encephalopathy and was transitioned to comfort-focused care; she died on hospital day 21.

We hypothesize that impaired gastric decompression due to the esophageal occlusion balloon of the LT, combined with repeated increases in intra-abdominal pressure during chest compressions, may have contributed to mucosal laceration at the esophagogastric junction in the absence of vomiting. However, because this is a single case, a causal relationship cannot be established, and this mechanism remains purely hypothesis-generating. This report highlights the need to consider MWS in the differential diagnosis of unexplained anemia or upper gastrointestinal bleeding after CPR, particularly when an LT has been used, and supports prompt evaluation even in the absence of antecedent emesis.

## Introduction

Mallory-Weiss syndrome (MWS) is a well-recognized cause of nonvariceal upper gastrointestinal bleeding (UGIB), accounting for approximately 3-10% of cases. First described in 1929, MWS is characterized by superficial, longitudinal mucosal lacerations at the esophagogastric junction, classically triggered by forceful vomiting, retching, or coughing [1]. Although MWS is classically preceded by forceful vomiting, it can occur without emesis when other mechanisms cause sudden increases in intragastric pressure [2]. Cardiopulmonary resuscitation (CPR)-related gastrointestinal complications, including gastric perforation and solid organ injury, have been documented in the literature [3], and CPR-associated MWS, while rare, has been reported in a small number of cases [4,5,6].

The laryngeal tube (LT) is a supraglottic airway device (SGA) that establishes a patent airway via a pharyngeal cuff and an esophageal occlusion balloon. In the Pragmatic Airway Resuscitation Trial (PART), a multicenter randomized controlled trial (n = 3,004), an LT-first strategy was associated with significantly greater 72-hour survival compared with endotracheal intubation (ETI) in out-of-hospital cardiac arrest (OHCA), supporting its role as a frontline prehospital airway device [7]. However, the esophageal occlusion balloon of the LT may obstruct gastric decompression pathways during CPR, a consequence that has not previously been examined in relation to MWS.

We report a case of MWS diagnosed without antecedent vomiting following LT-assisted CPR in a 77-year-old woman with no history of gastrointestinal disease, alcohol use, or other established MWS risk factors. To our knowledge, this is the first case in which the esophageal occlusion balloon of the LT has been proposed as a possible contributing structural factor in CPR-associated MWS. We discuss a plausible mechanism and its implications for post-resuscitation evaluation.

## Case presentation

A 77-year-old woman with a history of cerebral infarction (medications unknown) and no history of alcohol use or gastrointestinal disease called emergency medical services (EMS) for respiratory distress. She became unresponsive during the call. EMS arrived at 17:43 and found the patient deeply comatose, with a palpable but bradycardic radial pulse and jugular venous distension. After gaining entry to the residence, oxygen was administered via face mask at 10 L/min, and the patient was transferred to the ambulance. At 18:00, respiratory deterioration prompted the initiation of assisted bag-valve-mask (BVM) ventilation. Asystolic cardiac arrest was confirmed at 18:10, and chest compressions were initiated immediately.

After approximately 15 minutes of BVM ventilation (18:00-18:15), a size-3 LT (Smiths Medical Japan, Tokyo, Japan) - a standard LT without a gastric drainage lumen - was inserted at 18:15. Peripheral intravenous access was established, and a total of four doses of epinephrine (1 mg each, at 18:25, 18:29, 18:33, and 18:37) were administered. During resuscitation, the rhythm changed to pulseless ventricular tachycardia (VT), for which defibrillation shocks were delivered at 18:17 and 18:31, with intervening periods of asystole and pulseless electrical activity. Return of spontaneous circulation (ROSC) was achieved at 18:39. The total CPR duration from confirmed cardiac arrest to ROSC was approximately 29 minutes, during which the LT remained in place for approximately 24 minutes.

On hospital arrival, the Glasgow Coma Scale (GCS) score was 3 (E1V1M1) with a blood pressure of 89/56 mmHg, body temperature of 34.4°C, and respiratory rate of 14 breaths/min. Laboratory findings, summarized in Table 1, showed severe metabolic acidosis (pH 7.117; lactate 13.2 mmol/L), markedly elevated cardiac biomarkers (troponin I, 288 pg/mL; B-type natriuretic peptide (BNP), 792.2 pg/mL), hypoalbuminemia (albumin 2.7 g/dL), prolonged activated partial thromboplastin time (APTT, 71.2 sec), and elevated D-dimer (26.8 μg/mL). Admission hemoglobin was 11.3 g/dL. The patient had no documented history of vomiting, hematemesis, melena, alcohol use, or gastrointestinal disease. EMS personnel reported no emesis or vomitus on the patient's clothing or at the scene.

**Table 1:** Laboratory data on admission Laboratory data obtained on admission to the emergency department AST: aspartate aminotransferase; ALT: alanine aminotransferase; LDH: lactate dehydrogenase; CK: creatine kinase; CK-MB: creatine kinase-MB isoenzyme; BUN: blood urea nitrogen; WBC: white blood cell count; PT-INR: prothrombin time-international normalized ratio; APTT: activated partial thromboplastin time; BNP: B-type natriuretic peptide; PaCO₂: partial pressure of arterial carbon dioxide; PaO₂: partial pressure of arterial oxygen; HCO₃⁻: bicarbonate

Parameter	Value	Reference range
Biochemistry		
AST	24	13–33 U/L
ALT	15	8–42 U/L
LDH	246	124–222 U/L
CK	198	55–215 U/L
CK-MB	21.8	<25 U/L
BUN	14.3	8–20 mg/dL
Creatinine	0.71	0.65–1.07 mg/dL
Uric acid	5.6	2.5–7.0 mg/dL
Glucose	202	70–109 mg/dL
Sodium	140	138–145 mEq/L
Potassium	3.9	3.6–4.8 mEq/L
Albumin	2.7	4.1–5.1 g/dL
Complete blood count		
WBC	13,200	3,300–8,600/μL
Hemoglobin	11.3	11.6–14.8 g/dL
Platelets	18.7	15.8–34.8 ×10⁴/μL
Coagulation/hemostasis		
PT-INR	1.17	0.85–1.15
APTT	71.2	25.0–38.0 sec
Fibrinogen	268	200–400 mg/dL
D-dimer	26.8	<1.0 μg/mL
Biomarkers		
Troponin I	288	<26.2 pg/mL
BNP	792.2	<18.4 pg/mL
Arterial blood gas		
pH	7.117	7.35–7.45
PaCO₂	42.1	35–45 mmHg
PaO₂	60.3	80–100 mmHg
HCO₃⁻	13.3	22–26 mmol/L
Lactate	13.2	0.5–1.6 mmol/L

Table 2 presents the clinical timeline from EMS contact through hospital course.

**Table 2:** Clinical timeline Clinical timeline from EMS contact through hospital course EMS: emergency medical services; JCS: Japan Coma Scale; JVD: jugular venous distension; O₂: oxygen; BVM: bag-valve-mask; CPR: cardiopulmonary resuscitation; LT: laryngeal tube; VT: ventricular tachycardia; DC: direct current (defibrillation); IV: intravenous; PEA: pulseless electrical activity; ROSC: return of spontaneous circulation; BP: blood pressure; GCS: Glasgow Coma Scale; CT: computed tomography; NG: nasogastric; MWS: Mallory-Weiss syndrome; pRBC: packed red blood cells; FFP: fresh frozen plasma; Hb: hemoglobin; MRI: magnetic resonance imaging

Time	Event	Details
17:43	EMS contact	JCS 300; bradycardic but palpable radial pulse; JVD; no vomitus on clothing or at scene
17:43–18:00	Scene management	Entry to residence; O₂ via face mask at 10 L/min; transfer to ambulance; pulse still palpable
18:00	Respiratory deterioration	Assisted BVM ventilation was initiated due to respiratory deterioration
18:10	Cardiac arrest confirmed	Asystole; chest compressions initiated (CPR start)
18:15	LT inserted	Size-3 LT (Smiths Medical Japan); assisted BVM ventilation (18:00–18:10) followed by CPR ventilation (18:10–18:15); total BVM approximately 15 min
18:17	Pulseless VT → DC ①	First defibrillation
18:19	Asystole	
18:24	IV access	Right radial vein, 22G
18:25	Asystole + Epinephrine ①	1 mg IV
18:29	PEA + Epinephrine ②	1 mg IV
18:31	Pulseless VT → DC ②	Second defibrillation
18:33	Asystole + epinephrine ③	1 mg IV
18:35	Asystole	
18:37	Asystole + epinephrine ④	1 mg IV
18:39	ROSC	Total CPR duration (18:10–18:39): approximately 29 min; LT in place approximately 24 min
Post-ROSC	Hospital arrival	BP 89/56 mmHg; GCS 3 (E1V1M1); temp 34.4°C; severe metabolic acidosis
Post-ROSC	Contrast-enhanced CT	Active extravasation into the gastric lumen (Figure 1)
Post-CT	NG tube insertion	Bloody aspirate; inserted after CT confirmation of bleeding
Post-CT	Emergency endoscopy	Longitudinal mucosal laceration at the gastric cardia; MWS (Figure 2)
Post-scope	Endoscopic hemostasis	4 units pRBC + 4 units FFP; Hb recovered to 12.8 g/dL
Hospital day 5	Neurological assessment	Absent corneal/pupillary reflexes; brain MRI: diffuse hypoxic-ischemic injury
Hospital day 5	Goals of care discussion	Transitioned to comfort-focused care
Hospital day 21	Patient death	

Because of hemodynamic instability and a suspicion of internal bleeding, contrast-enhanced CT was performed, which demonstrated active extravasation of contrast into the gastric lumen (Figure 1). An NG tube was subsequently inserted and yielded a bloody aspirate. Because imaging preceded NG tube placement, the mucosal lesion was already present before tube insertion, making iatrogenic injury from the NG tube unlikely.

**Figure 1:**
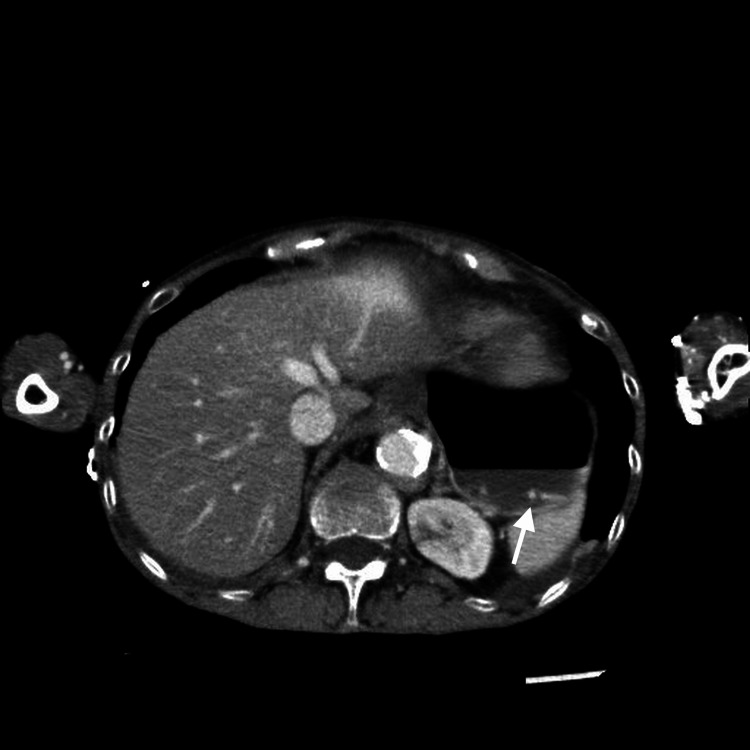
Contrast-enhanced CT demonstrating active extravasation into the gastric lumen Contrast-enhanced CT (venous phase) demonstrating active extravasation of contrast material into the gastric lumen (arrow), indicating active bleeding at the gastric cardia. This finding was identified before nasogastric tube insertion, supporting the interpretation that the mucosal injury was pre-existing and not caused by nasogastric tube placement CT: computed tomography

Emergent upper gastrointestinal endoscopy identified a longitudinal mucosal laceration at the gastric cardia (Figure 2), consistent with MWS and confirming the diagnosis. Endoscopic hemostasis was achieved successfully. Following ICU admission, the hemoglobin level declined to 8.9 g/dL before hemostasis but recovered to 12.8 g/dL after transfusion of four units of packed red blood cells and four units of fresh frozen plasma.

**Figure 2:**
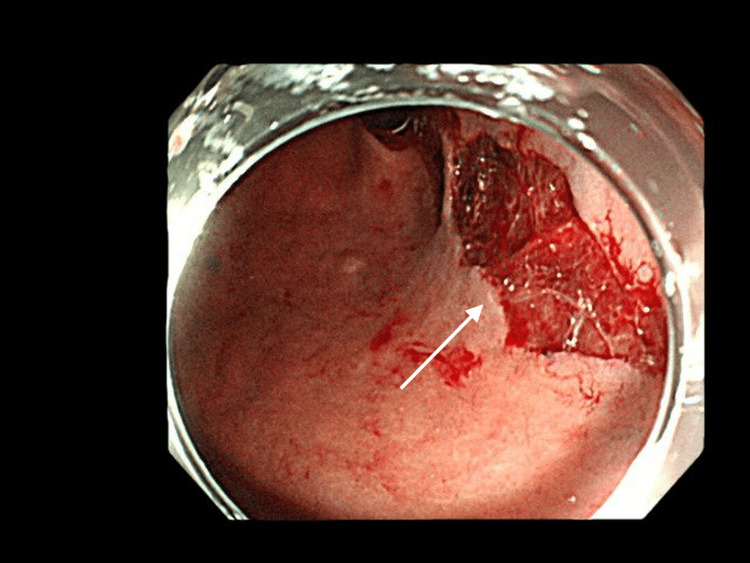
Upper gastrointestinal endoscopic image showing MWS Emergent upper gastrointestinal endoscopy showing a longitudinal mucosal laceration at the gastric cardia (arrow), consistent with MWS. The laceration is located at the esophagogastric junction. The diagnosis was confirmed after contrast-enhanced CT demonstrated active extravasation into the gastric lumen, followed by nasogastric tube insertion yielding a bloody aspirate. Endoscopic clipping was used to achieve hemostasis MWS: Mallory-Weiss syndrome; CT: computed tomography

Despite successful hemostasis, the patient developed severe hypoxic-ischemic encephalopathy. On hospital day five, corneal and pupillary reflexes remained absent, and brain MRI demonstrated diffuse restricted diffusion consistent with diffuse hypoxic-ischemic injury. After a detailed discussion with the family regarding the poor neurological prognosis, the patient was transitioned to comfort-focused care, and she died on hospital day 21.

## Discussion

Atypical presentation of MWS

Although MWS classically presents with hematemesis after forceful vomiting, it can occur without antecedent emesis [2]. In a large series of 2,175 patients with upper gastrointestinal bleeding, Sugawa et al. identified MWS in 224 cases (10.3%), noting that the condition was self-limiting in over 90% of patients [2]. A subsequent systematic review and meta-analysis by Albalawi et al. identified hiatal hernia as the most significant risk factor for MWS (OR: 1.96, 95% CI: 1.73-2.22), while alcohol use was not found to be a strong predictor. Neither a hiatal hernia nor chronic alcohol use was present in our patient [8]. In our patient, the diagnosis was established through CT findings obtained for evaluation of hemodynamic instability, highlighting the need for a heightened index of suspicion for MWS in post-resuscitation patients who present with unexplained anemia or hemodynamic instability.

Possible mechanism: LT use during CPR

Spoormans et al. reviewed 67 cases of gastric perforation following CPR and identified gastric overdistension from excessively forceful or rapid rescue breathing as the principal contributing factor [3]. Two factors may have acted in concert in the present case. First, air introduced during the prolonged BVM ventilation phase (approximately 15 minutes) - an established contributor to CPR-related gastric injury [3] - likely caused some initial gastric distension before LT insertion. Second, and central to our hypothesis, the LT's esophageal occlusion balloon seals the proximal esophagus and thereby obstructs the natural pathway for gastric decompression once mechanical ventilation continues. Unlike second-generation SGAs such as the laryngeal mask airway (LMA) ProSeal or i-gel, which incorporate a dedicated gastric drainage channel [9], the LT does not allow active gastric venting; intragastric pressure may therefore rise as ventilation continues.

Mann et al. have documented substantial gastric air distension during LT-assisted mechanical ventilation, lending physiological plausibility to this concern [10]. Repeated chest compressions further generate cyclic increases in intra-abdominal and intragastric pressure. We hypothesize that the combination of impaired gastric decompression from the occlusion balloon and CPR-induced pressure surges may have generated an intragastric pressure sufficient to cause mucosal laceration at the esophagogastric junction. This interpretation, however, remains speculative. Because this is a single case, causality cannot be established, and alternative contributors - particularly the prolonged BVM ventilation phase and the intra-abdominal pressure changes generated by chest compressions themselves - remain plausible and cannot be disentangled from the proposed role of the laryngeal tube. The relative contribution of each factor cannot be quantified from a single observation, and our proposed mechanism should therefore be regarded as hypothesis-generating rather than established.

Excluding the nasogastric tube as a cause

An important consideration is whether NG tube insertion could have caused the mucosal laceration. Contrast-enhanced CT demonstrated active extravasation into the gastric lumen before the NG tube was inserted; the tube subsequently yielded bloody aspirate. This temporal sequence indicates that the mucosal laceration was present before NG tube placement, making iatrogenic injury unlikely. Furthermore, NG tube-related esophagogastric injuries typically present as circumferential or punctate lesions rather than the longitudinal laceration characteristic of MWS, further arguing against the NG tube as a causative factor.

Conceptual device comparison and clinical implications

Table 3 presents a conceptual comparison of airway devices based on their structural design features and relevant literature, not on head-to-head clinical data. The LT's esophageal occlusion balloon distinguishes it from other SGAs in that it lacks a gastric drainage channel, a design feature that may theoretically limit intragastric pressure relief during CPR. This structural difference, supported by Mann et al.'s observation of gastric air distension during LT-assisted ventilation [10], forms the basis of our hypothesis. No direct comparative data exist for gastrointestinal complication rates across SGA types, and this table should be interpreted as a hypothesis-generating framework rather than an evidence-based risk ranking.

**Table 3:** Conceptual comparison of airway devices based on design features and literature This table presents a conceptual comparison based on device design features and the available literature, not head-to-head clinical data. No comparative clinical studies have established a definitive risk hierarchy for gastrointestinal complications across these devices during cardiopulmonary resuscitation. This table should be interpreted as a hypothesis-generating framework rather than as evidence-based risk stratification ETT: endotracheal tube; LT: laryngeal tube; LT-D: laryngeal tube with drainage; LT-S: laryngeal tube suction; LMA: laryngeal mask airway; BVM: bag-valve-mask; ALS: advanced life support; PART: Pragmatic Airway Resuscitation Trial; CPR: cardiopulmonary resuscitation

Feature	ETT	LT	LMA ProSeal/i-gel	BVM
Esophageal seal	None (tracheal seal)	Yes (esophageal occlusion balloon)	Partial (perilaryngeal seal)	None
Gastric drainage channel	N/A	No (standard LT); newer LT-D/LT-S variants have drainage port	Yes (dedicated gastric channel)	None
Pathway for gastric venting	Open (esophagus unobstructed)	Obstructed by an occlusion balloon	Open via drainage channel	Open (esophagus unobstructed)
Risk of gastric insufflation	Low	Theoretical: possible during prolonged use	Reduced (active venting available)	High (well-documented)
Prehospital ease of insertion	Difficult (requires laryngoscopy)	Easy (blind insertion)	Easy (blind insertion)	Easy (no airway device)
Use in CPR per major guidelines	Endorsed by ALS guidelines	Endorsed (PART trial evidence)	Endorsed in select settings	First-line before advanced airway

The PART trial demonstrated that an LT-first strategy was associated with significantly greater 72-hour survival than an ETI-first strategy in OHCA (18.3% vs. 15.4%; adjusted difference: 2.9%, 95% CI: 0.2%-5.6%; p = 0.04) [7]. The established survival benefit of LT-first prehospital management must therefore be weighed against the present hypothesis. Our case raises a hypothesis, not a demonstrated risk, that the LT's esophageal occlusion balloon may merit consideration in post-resuscitation monitoring.

Post-resuscitation airway management considerations

In our patient, the absence of antecedent vomiting meant that MWS would not have been suspected clinically; the diagnosis was established only because contrast-enhanced CT was performed for the evaluation of hemodynamic instability. This experience suggests that clinicians caring for post-resuscitation patients who received LT-assisted CPR should maintain a low threshold for upper gastrointestinal evaluation, including gastric aspiration, contrast-enhanced imaging, and endoscopy, when unexplained hypotension or progressive anemia is observed, even when antecedent vomiting is absent. In Japan, standard laryngeal tubes that lack a gastric drainage channel are frequently used by prehospital emergency medical services alongside newer variants that incorporate a drainage port. In settings where such devices predominate, early conversion from LT to endotracheal intubation upon hospital arrival may help restore the natural gastric decompression pathway and limit ongoing gastric insufflation. We emphasize that this is a hypothesis derived from a single case and requires prospective evaluation before any change in clinical practice can be recommended.

Comparison with previously reported cases

Norfleet and Smith reported MWS after successful CPR in 1990, noting that only three similar cases had been described in the literature in the English language at that time [4]. Kim et al. subsequently reported a fatal case of CPR-associated MWS with an estimated blood loss of 3,000 mL [5]. Yu et al. described MWS following CPR for acute myocardial infarction, attributing the mechanism to elevated intragastric pressure caused by chest compressions and rescue breathing [6]. Tang et al. reported MWS after CPR combined with transesophageal echocardiography, suggesting that esophageal instrumentation during resuscitation may potentiate mucosal injury [11]. None of these prior reports identified a specific airway-device feature as a contributing factor. Our case extends this literature by raising the possibility that the LT's esophageal occlusion balloon represents a previously unrecognized, device-specific contributor to CPR-associated MWS.

Limitations

This report has several limitations. First, as a single case, it precludes causal inference; the relative contribution of the LT versus other factors, including the initial BVM ventilation phase, which itself can cause gastric overdistension [3], cannot be quantified. Second, the patient's profound unconsciousness precluded a complete prehospital history; her home medications were unknown, so the contribution of antiplatelet, anticoagulant, or nonsteroidal anti-inflammatory drugs to bleeding diathesis could not be formally excluded, although laboratory coagulation parameters (PT-INR 1.17) did not indicate clinically significant anticoagulation. Although no vomitus was observed on the patient's clothing or at the scene by EMS personnel, unwitnessed vomiting before EMS arrival cannot be entirely excluded.

Third, the underlying cause of cardiac arrest could not be definitively established; the presentation with respiratory distress, jugular venous distension, and elevated BNP suggests a cardiac etiology, but this remained presumptive. Fourth, although cardiac arrest was identified by EMS personnel on scene and chest compressions were initiated without delay, the patient had been found deeply comatose with a bradycardic but palpable pulse upon EMS arrival at 17:43. Respiratory deterioration requiring BVM ventilation occurred at 18:00, followed by confirmed asystolic arrest at 18:10. The duration and degree of circulatory compromise during this pre-arrest interval could not be precisely quantified. Fifth, our hypothesis applies specifically to the standard LT used in this case, which lacks a gastric drainage lumen; newer variants such as the LT-D and LT-S (LTS-D) incorporate a gastric drainage channel and may not carry the same theoretical risk, a distinction that requires further investigation. Sixth, Table 3 provides a conceptual summary based on device design and the available literature; no head-to-head comparative data exist for gastrointestinal complication rates across SGA types during CPR, and the table should not be interpreted as establishing a definitive risk hierarchy. Prospective multicenter surveillance is needed to determine the true incidence of MWS following LT-assisted CPR.

## Conclusions

We described a rare case of MWS occurring without antecedent vomiting after CPR with an LT. To our knowledge, this is the first case in which the esophageal occlusion balloon of the LT has been proposed as a possible contributing structural factor. Impaired gastric decompression caused by the occlusion balloon, combined with repeated increases in intra-abdominal pressure during chest compressions, may have contributed to mucosal laceration at the esophagogastric junction. The diagnosis was established by CT before NG tube insertion, making iatrogenic injury from tube placement unlikely. Clinicians should consider MWS in the differential diagnosis of unexplained UGIB after resuscitation, particularly when an LT has been used. When bleeding is suspected, prompt evaluation with gastric aspiration, contrast-enhanced imaging, and endoscopy is warranted even in the absence of antecedent vomiting. In settings where LTs without a gastric drainage channel are frequently used as the standard prehospital airway device, early conversion to ETI after hospital arrival may warrant consideration to restore the gastric decompression pathway.
